# Building Resilience Through Better Performance Assessment of Switzerland’s Health System in Times of Crises

**DOI:** 10.3389/phrs.2025.1608860

**Published:** 2025-12-15

**Authors:** Camille Poroes, Laurence Seematter-Bagnoud, Kaspar Wyss, Isabelle Peytremann-Bridevaux

**Affiliations:** 1 Department of Epidemiology and Health Systems, Unisanté, University Center for Primary Care and Public Health & University of Lausanne, Lausanne, Switzerland; 2 Swiss Centre for International Health, Swiss Tropical and Public Health Institute, Allschwil, Switzerland; 3 Faculty of Science, University of Basel, Basel, Switzerland

**Keywords:** health system, performance, resilience, crisis, assessment

## Abstract

**Background:**

The increase in patients’ needs and demands resulting from the COVID-19 pandemic has led to an assessment of whether health systems were performant enough. Health system performance refers as how far health systems achieve their desired goals. Measuring the Swiss health system performance taking into account its ability to prepare, manage and learn from a crisis will allow to better interpret the assessment.

**Analysis:**

Assessments of the Swiss health system performance appear not to fully align to recent developments on conceptual thinking of the WHO and the OECD, notably regarding the assessment of the resilience of a health system, and need to be modified to better reflect current developments.

**Policy Options:**

Recommendations include considering resilience as a core concept, standardizing a framework integrating resilience, considering resilience indicators, and enhancing data collection and sharing.

**Conclusion:**

To ensure long-term resilience and performance, Switzerland must act decisively to unify its data systems, institutionalize regular performance reviews including resilience indicators, and build a common framework and language for resilience.

## Background

Any type of crisis (environmental, financial, social or sanitary, for example) can profoundly impact health systems and the delivery of routine medical and public health services. A crisis can be characterized by a period of difficulty, danger or uncertainty, usually caused by unexpected events that perturb the usual functioning of healthcare facilities and can be declined in four different phases: 1. Preparedness, 2. Shock onset and alert, 3. Shock impact and management, 4. Recovery and learning [1].

In Switzerland, as elsewhere, the COVID-19 pandemic and its consequences such as higher morbidity and mortality rates have placed a considerable challenge on the entire health system [2]. Contingency measures in 2020 resulted in a drop of hospital admissions (−5%) and a decline in the number of nursing homes admissions (−3%). At the same time, the number of deaths increased significantly in both hospitals (+8%) and nursing homes (+16%). Between 16 March and 5 April 2020, hospitalizations accounted for 51% more hours in intensive care than during the same period in previous years [3]. The increase in patients’ needs and demands resulting from the pandemic led to an assessment of whether health systems were performant enough to meet these challenges [4].

This highlighted the critical need for health systems to have the capacity to maintain effectiveness, efficiency, equity, and responsiveness in the face of high-impact shocks. In this context, the development of ways for assessing their performance in times of crisis has become a matter of concern. The World Health Organization (WHO) stated in 2007 that health system performance refers as how far health systems achieve their desired goals, such as: to improve the health of the population, to respond to the reasonable expectations of the population, and to provide financial protection against the costs of ill-health [5]. However, there is currently no universally accepted method for assessing it in times of crisis.

Measuring the Swiss health system performance taking into account its ability to prepare, manage and learn from a crisis [6] will allow to better interpret the assessment, to identify where the health system is facing challenges, and explore possible solutions for improvement to reach citizens health, population expectations and the financial protection.

## Analysis

One of the main tools currently used to assess the performance of a health system is the Health System Performance Assessment (HSPA) proposed by the WHO in 2012. It evaluates the health system as a whole, linking health outcomes to strategies or functions using a limited number of quantitative indicators [7]. Although a crisis can have a major impact on the system itself, this tool has long been not adapted to a time of crisis, since it did not consider the components of a crisis.

**FIGURE 1:**
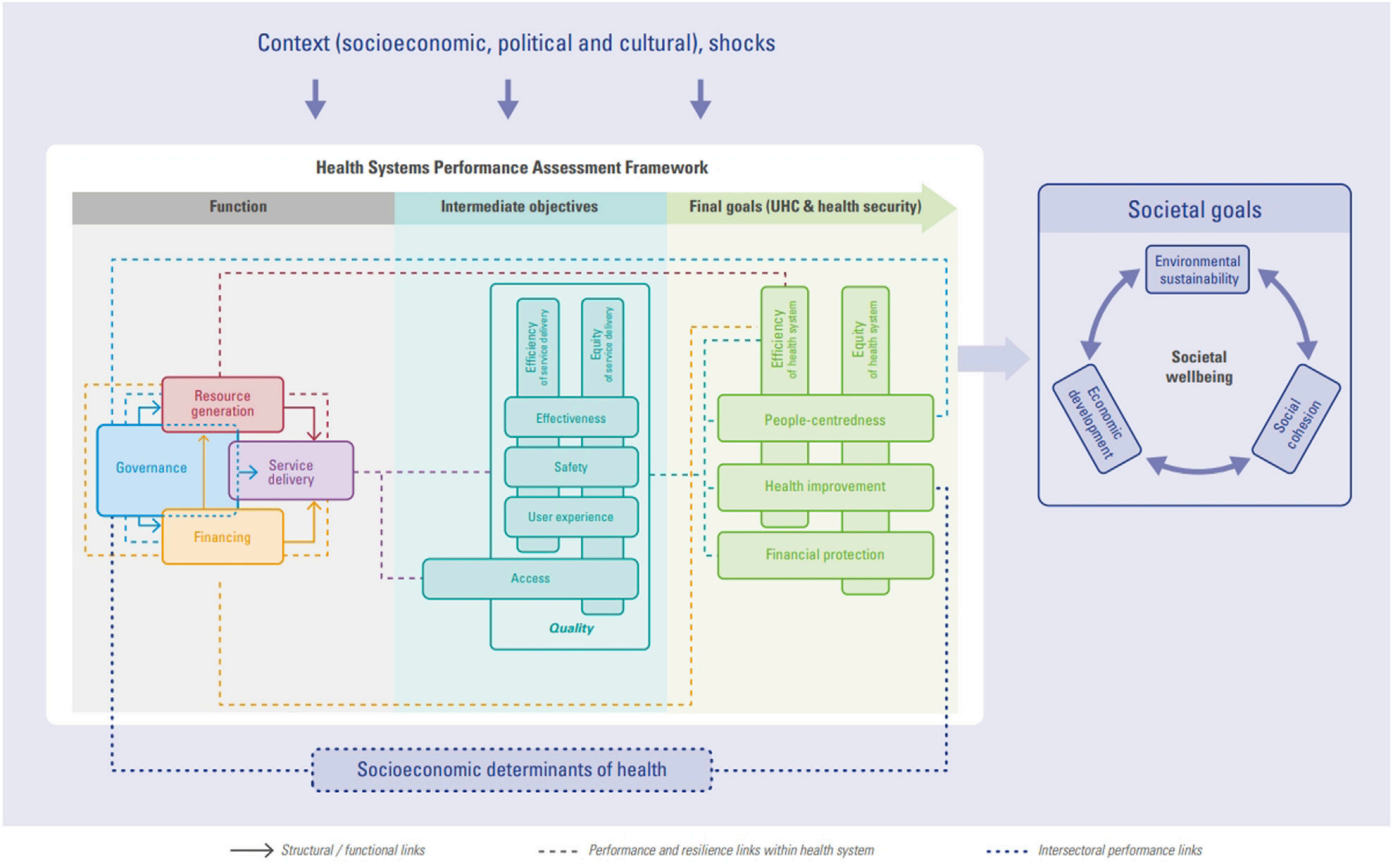
Health system performance assessment framework by WHO/European Observatory on health systems and policies (A practical Handbook for resilience testing, Paris, France, 2024) [9].

The COVID-19 pandemic revealed that many health systems were underprepared and not as resilient as previously thought, leading to increased emphasis on assessing and building health system resilience to future shocks [8]. Thus, in March 2024, the European Observatory on Health Systems and Policies (hosted by WHO) published a practical handbook for resilience testing [9], in which they proposed to adapt the HSPA framework for using it in times of crisis by including the notion of resilience (Figure 1). They defined health systems resilience as “how well the key health system functions perform in the face of shocks, and therefore the extent to which the system as a whole can continue to meet its intermediate and final objectives.” They argue that the “HSPA Framework serves as a conceptual framework that allows for systematic assessment of resilience that considers each individual part of the health system.” The Organisation for Economic Co-operation and Development (OECD) also recently updated its HSPA framework to integrate new dimensions of performance such as resilience, people-centeredness, and environmental sustainability [4]. Combining the notions of resilience and performance through a joint framework is one of the first steps to adapt the assessment of health system performance in times of crisis. However, these frameworks and new vision of integrating both notions together are newly emerging and not widely used yet.

There are several studies to assess performance of the Swiss health system relative to those of other countries, such as the OECD publication Health at a Glance, the Commonwealth Fund’s International Health Policy Surveys, and the European Observatory on Health Systems and Policies’s “Health Systems and Policy Monitor (HSPM)” and “Health Systems in Transition (HiT) series” (new version planned for Switzerland mid 2026) [10]. However, none of these studies placed a particular emphasis on measuring the performance when facing a crisis. i.e., resilience.

Given that context, assessments of the Swiss health system performance appear not to fully align to recent developments on conceptual thinking of the WHO and the OECD and need to be modified to better reflect current developments.

## Policy Options

**FIGURE 2:**
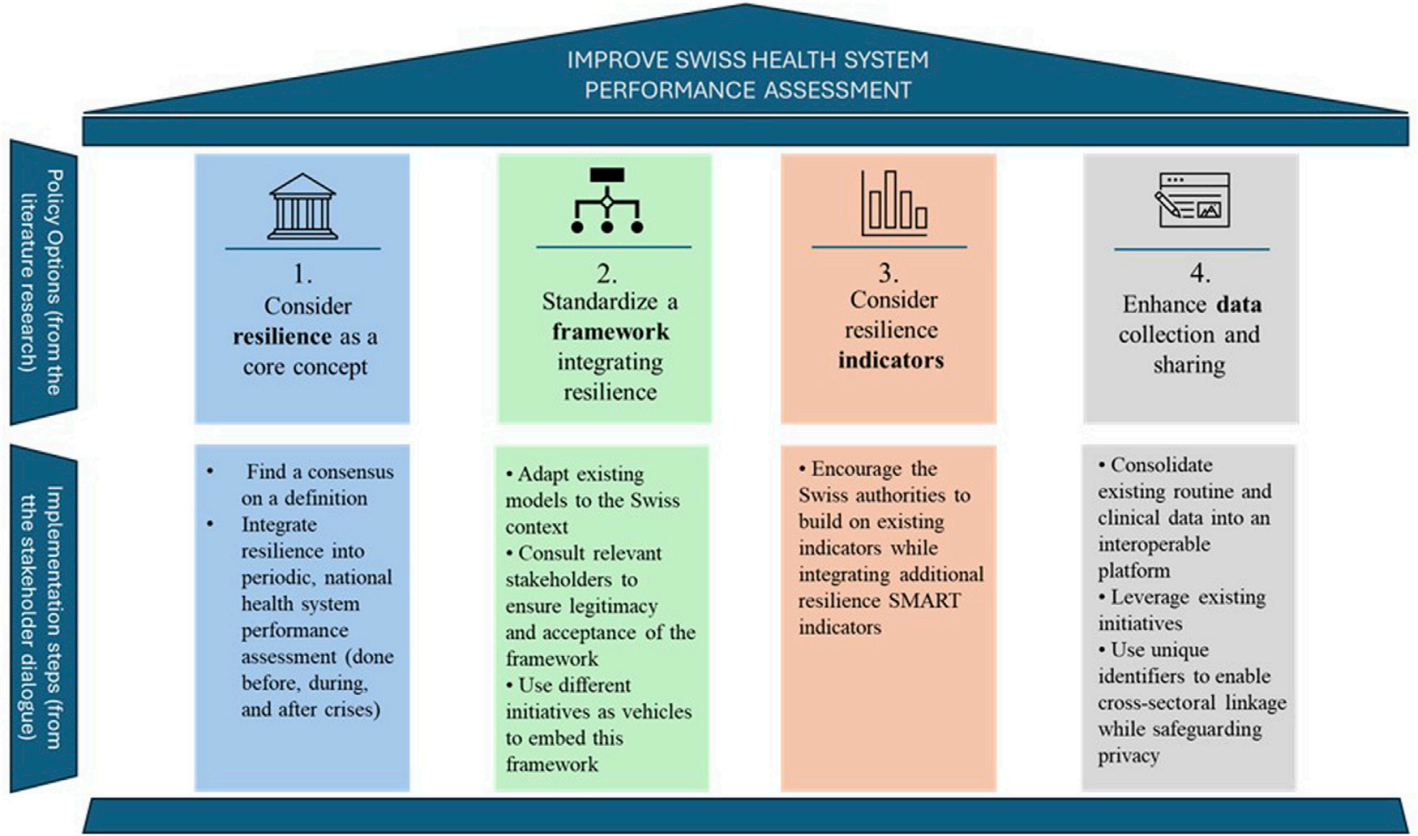
Key policy options and implementation steps to improve the Swiss Health System Performance Assessment (Lausanne, Switzerland, 2025).

In order to respond to the concern of assessing and monitoring the performance of the Swiss health system in times of crisis, we present four recommendations that could be implemented in Switzerland (Figure 2).

These recommendations are based on the findings of a comprehensive review of existing literature on health system performance and resilience. This review built upon a prior literature search using PubMed in May 2021 and updated in April 2023 that focused on health system performance and resilience frameworks [11]. This search strategy focused on international, system-wide frameworks published in English or French since 2005. Publications were screened and selected through a two-step process using Covidence [12], with validation by two reviewers. The findings from this prior literature search, which have already been published [11], served as the foundation for this policy brief, and were then updated for inclusion of publications up to 2025, ensuring the inclusion of the newly emerging and relevant literature.

In addition, international grey literature was reviewed to identify comprehensive approaches for assessing health system performance and resilience. Also, Swiss official documents and other relevant national publications were included to contextualize findings within the Swiss health system.

The findings from this literature research led to the identification of four recommendations that were further informed by insights from a stakeholder dialogue conducted in March 2025. The eight stakeholders were recruited to ensure diverse representation from key institutions, including the Swiss Tropical and Public Health Institute (Swiss TPH), Association suisse des infirmières et infirmiers SBK-ASI, Swiss Learning Health System (SLHS), Unisanté, the European Observatory on Health Systems and Policies, and the Obsan. The dialogue relied on structured guidance and was led by a moderator and supported by two facilitators, using a combination of collaborative problem-solving, participatory learning, and structured decision-making methods. All discussions were documented and thematically analyzed to prioritize recommendations and identify context-specific solutions aligned with Swiss health system.

### Option 1: Consider Resilience as a Core Concept

The ability of a health system to anticipate, manage, adapt to and learn from sudden and severe disruptions [1] refers to its resilience. The term resilience was mainly used in fields such as psychology, ecology, engineering and materials science [13, 14] and arose in health systems research following crises such as Ebola in West Africa in 2014 and the recent COVID-19 pandemic [8, 15–17].

A resilient health system is essential to ensure continuity in maintaining essential health functions in the event of a crisis, and thus guarantee the continuity of its operations [18]. A resilient response to a shock implies the implementation of strategies that maintain the functioning of health systems and preserve overall performance [6].

As the notions of performance and resilience cannot be separated in times of crisis, it would be worth to combine both concepts and track them over time to understand how health systems resist to crises and help them cope adequately with future shocks. Moreover, aligning the concepts of performance and resilience for the assessment of health systems was recently supported by the WHO in their work in progress on “Improving the performance and resilience of health systems” [19].

The first option addressed to Swiss decision-makers would then be to consider resilience as an inherent notion of health system performance assessment. For this purpose, consensus on a definition of resilience is needed in Switzerland. Then, resilience should be integrated into periodic, national health system performance assessment, and the monitoring of health system performance should be done before, during, and after crises (Figure 2).

### Option 2: Standardize a Framework Integrating Resilience

**FIGURE 3:**
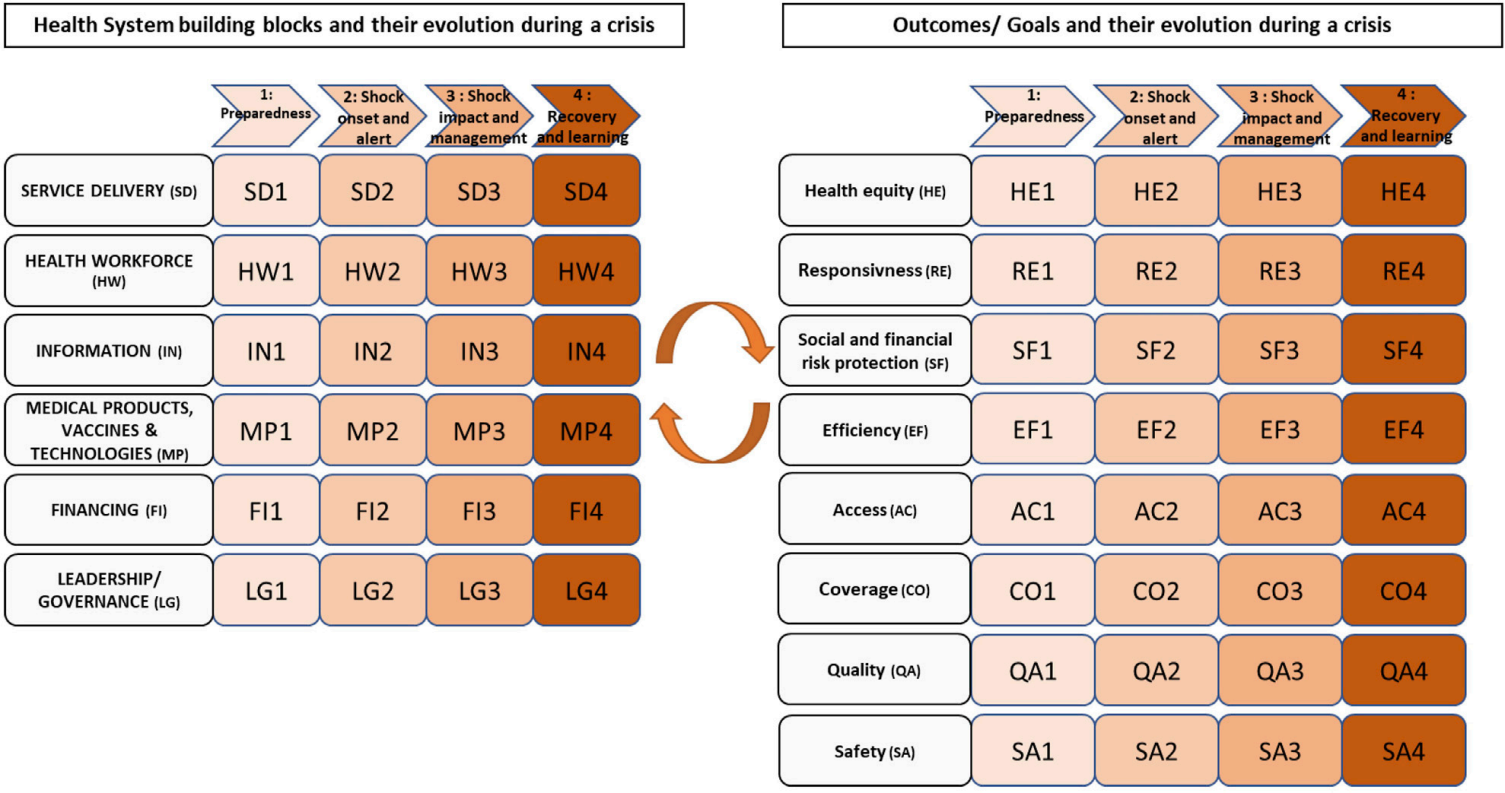
Health system performance and resilience framework by Poroes et al. (Health System Performance and Resilience in Times of Crisis: An Adapted Conceptual Framework, Lausanne, Switzerland, 2023 [11].

To develop the idea of the first recommendation further, we have proposed an adapted framework that combines both notions of performance and resilience (Figure 3) [11]. This model is a combination of the well-known WHO performance model, the Six-Building Blocks model (including service delivery, health workforce, information, medical products, vaccines and technologies, financing, and leadership/governance) [20] and the resilience model proposed by Thomas et al. [1], the latter conceptualizing the phases of a crisis. In this adapted framework, the six building-blocks are organized according to the four phases of a crisis (preparedness, shock onset and impact, shock management, and learning/recovery). In this way, the performance and resilience evaluation are adapted according to the phase of the crisis in which the system finds itself. More details and explanations about this framework can be found in this Ref. [11].

Adapted conceptual frameworks, such as the ones from the European Observatory on Health Systems and Policies [21] and the OECD [4] or the one from our recent work (Figure 3) [11], enable a common language, systemic analysis of health system functions and more useful performance evaluation results for policymakers. The adoption of one of these frameworks as one’s standard could enable the policymakers to find reliable systematic and standardized information on the design and functioning of a health system and comparable information on other countries’ health systems in times of crisis. Moreover, it can enhance understanding of the Swiss health system characteristics, strengths and limitations for evaluators, researchers, and public health practitioners [22].

To institutionalize such a framework in Switzerland, a feasible approach would be to designate an independent institution, such as the Swiss Health Observatory (Obsan) or the Federal Office of Public Health (FOPH), to lead and coordinate implementation. The agency mandate with the oversight on the framework could define methodological standards for resilience assessment and ensure coherence and transparency in framework application. To operationalize this role, the conference of the cantonal health directors (CDS/GDK) agreements would be required to align evaluation criteria across the federal and cantonal levels.

Practical actions to implement this option could be to adapt existing models to the Swiss context. The Swiss decision-makers can learn from EU countries that integrated resilience into their HSPA (e.g., Belgium) [23]. Relevant stakeholders could be consulted to ensure legitimacy and acceptance of the framework. Then, different initiatives such as Health 2030 (Federal Council’s health policy strategy 2020–2030) and the HIT reviews could be used as vehicles to embed this framework, to finally put at the agenda as a policy priority “health system resilience” (Figure 2).

### Option 3: Consider Resilience Indicators

In order to have a framework that is practical and to give concrete meaning to the options 1 and 2, it is important to identify appropriate resilience indicators. As emphasized by a systematic review on health system resilience metrics and indicators in high-income countries, measuring health system resilience is essential for understanding and building resilient health systems [24]. The consideration of indicators measuring the impact of challenges/crisis is necessary in all health system performance frameworks to ensure that the perspective of resilience is taken into account. This means to monitor and analyze the evolution of specific indicators regarding the performance and the resilience jointly. Therefore, a list of indicators regarding the performance and the resilience should be proposed, which should be based on an adapted framework as the one proposed in our recent work [11]. For example, on one hand, unmet healthcare needs and avoidable mortality could be two high-level indicators among others to assess the performance of a health system [25]. On the other hand, emergency workforce planning and emergency funds could be part of a resilience assessment [21].

In Switzerland, the FOPH and the Federal Statistical Office (FSO) work conjointly with the Obsan on health indicators. One of the goal of the Obsan is to produce and update a series of indicators on health and healthcare system, for the following themes: population health, mental health, age and long-term care, healthcare system, healthcare professionals, and costs and financing [26]. Implementation and collection of resilience and performance indicators could therefore build on existing processes and systems in place such as the Obsan. Data could also be sourced through existing mechanisms such as ANQ (the Competence Center for Quality Measurement in Hospitals and Clinics) or directly at the FSO. There is a need to develop a focused set of meaningful indicators, rather than relying on extensive but underutilized datasets. These indicators should meet the SMART criteria—Specific, Measurable, Achievable, Relevant, and Time-bound—and capture both routine operations and responses to crises. Furthermore, a foundational set of resilience indicators applicable to all types of crises should be established, supplemented by tailored indicator sets designed for specific crisis scenarios, such as pandemics, climate-related events, or financial disruptions (Figure 2). In Supplementary Material 1, we have provided an example shortlist of indicators for a Resilience-Based Health System Performance Assessment for Switzerland.

### Option 4: Enhance Data Collection and Sharing

Encouraging stakeholders to collect and share their data is key when aiming at assessing health system performance and resilience. A robust health system performance assessment is inextricably linked to data availability. Health-related data from various sources, such as registers, surveys, hospital data, and medical records, can provide valuable input for assessing health systems [27]. While measures of health inputs such as number of facilities may be broadly available, quality metrics and patient-reported outcomes are not [28].

Knowing that Swiss population (71%) is willing to share anonymized health data [29], further actions should be put in place to consolidate existing routine and clinical data into an interoperable platform. Building interoperability requires at least the adoption of unique patient and provider identifiers, and a standardized minimum data set for resilience and performance indicators. Leveraging the Swiss population’s high willingness to share anonymized health data, authorities could even strengthen more public trust by clearly communicating data protection safeguards and the tangible benefits of data use for health system improvement (Figure 2).

### Example Scenario: COVID-19

Swiss Vignette (What-if Case 2020–2022): Had the four proposed recommendations (policy options presented in this Policy Brief) been implemented before the COVID-19 pandemic, the Swiss population could have benefited from earlier and more targeted measures. For instance, continuous monitoring of Intensive Care Unit occupancy thresholds and staff absenteeism rates would have triggered early alerts in February–March 2020, encouraging early coordination between cantons and workforce reallocation before critical capacity was reached. Real-time tracking of primary care teleconsultation coverage could have identified unequal digital readiness across cantons, allowing authorities to support lagging regions with rapid telehealth deployment. Moreover resilience indicators, could have guided elective care prioritization, ensuring continuity for chronic patients and preventing avoidable morbidity. This vignette illustrates how a standardized, interoperable resilience framework could translate into faster, evidence-informed decisions, strengthening Switzerland’s capacity to manage future crises while maintaining essential health services. Table 1 complements the vignette by detailing how each recommendation can be deployed, ensuring that Resilience move from theory to practice in the Swiss health system context.

**TABLE 1:** Final Call-to-action Implementation roadmap according to the proposed recommendations (Lausanne, Switzerland, 2025).

Year	Recommendations	Next implementation steps for Switzerland
1	Consider resilience as a core concept	• Find a consensus on a health system resilience definition
2	Standardize a framework integrating resilience	• Establish a stakeholder task force under obsan/FOPH leadership
3	Consider resilience indicators	• Approve a core set of resilience indicators
4	Data collection and sharing	• Initiate pilot implementation in 2–3 cantons • Conduct the first national resilience review synthesizing pilot findings • Refine the framework based on lessons learned

## Conclusion

To ensure long-term resilience and performance, Switzerland must act decisively to unify its data systems, institutionalize regular performance reviews including resilience indicators, and build a common framework and language for resilience. By aligning relevant stakeholders on this topic – federal and cantonal actors-, the country can better prepare for future crises and turn monitoring into meaningful action.
