# Supplementary material for: Building Resilience Through Better Performance Assessment of Switzerland’s Health System in Times of Crises
Source: Public Health Rev. 2025 Dec 15;46:1608860. doi: 10.3389/phrs.2025.1608860 (PMC12745293; doi:10.3389/phrs.2025.1608860)
Supplement: Supplementary file 1 [file Table1.PDF]

Supplementary file 1 – Example shortlist of Indicators for a Resilience-Based Health System Performance Assessment (Switzerland)

#	Indicator	Definition	Numerator / Denominator	Primary Data Source/ Comments	Relevant Crisis Phases (1. Preparedness; 2. Shock onset and alert; 3. Shock impact and management; 4. Recovery and learning)
1	Percentage of the population going without a necessary healthcare service for financial reasons	Share of population reporting unmet medical needs	Percentage of the population in %	Federal Office of Public Health/ Obsan	All
2	Avoidable mortality rate	Deaths that could have been prevented through timely and effective care.	Number of avoidable deaths / total deaths (age-standardized)	Not found online for Switzerland	All
3	Rate of occupied hospital beds	Average daily number of occupied beds in acute somatic care	total number of care days divided by 365, per 1,000 inhabitants	Federal Statistical Office / Obsan	2; 3
4	Absences among nursing and care staff due to illness/accident	The indicator reflects the number of days per year during which care and support staff were absent from work due to illness or accident. The absenteeism rate is also provided, corresponding to the annual duration of absences as a percentage of the total annual working time defined by contract.	Number of full-time employee absence days and absence rate (%)	Federal Statistical Office / Obsan	2; 3

5	Staffing in nursing	This indicator reports the number of full-time equivalents (FTE) of care and support staff per 100 patients.	Care and support staff (in FTE) per 100 patients	Obsan/ This indicator does not assess staffing adequacy according to the situation, but offers a general overview and a basis for setting reference values	3
6	PPE stockout days	Days with reported shortages of essential protective equipment.	Days with stockout / total days	Not found online for Switzerland	2; 3
7	Primary care teleconsultation coverage	Share of primary care consultations delivered via telehealth.	Teleconsultations / total consultations	Not found online for Switzerland	2; 3
8	Excess mortality (all-cause)	Deviation of observed deaths from expected baseline.	Observed deaths – expected deaths	Not found online for Switzerland	2; 3
9	Time-to-data linkage	Average time between data collection and integration into national platform.	Days from data capture to integration	Not found online for Switzerland	All
